# New Insights into and Updates on Antimicrobial Agents from Natural Products

**DOI:** 10.1155/2019/7079864

**Published:** 2019-04-01

**Authors:** Taoufik Ghrairi, Sophie Jaraud, Artur Alves, Yannick Fleury, Allaeddin El Salabi, Chedly Chouchani

**Affiliations:** ^1^Laboratoire de Neurophysiologie, Physiopathologie Cellulaire et Valorisation des BioMolécules, Faculté des Sciences de Tunis, Université de Tunis El-Manar, Campus Universitaire Farhat Hachad, Tunisia; ^2^International Center for Infectiology Research (CIRI), Inserm, U1111, CNRS, UMR5308, ENS de Lyon, Université Claude Bernard Lyon 1, 69007 Lyon, France; ^3^Department of Biology, CESAM-Centre for Environmental and Marine Studies, University of Aveiro, Campus Universitário de Santiago, 3810-193 Aveiro, Portugal; ^4^Université de Bretagne Occidentale, LBCM EA, IUT Quimper, Quimper, France; ^5^Department of Environmental Health, Faculty of Public Health, University of Benghazi, Benghazi, Libya; ^6^Arab African Academy for Studies, Benghazi, Libya; ^7^Université de Carthage, Laboratoire de Recherche Sciences et Technologies de l'Environnement, ISSTE de Borj-Cedria, Technopôle de Borj-Cedria, BP 1003, Hammam-Lif 2050, Tunisia; ^8^Laboratoire des Microorganismes et Biomolécules Actives, Faculté des Sciences de Tunis, Université de Tunis El Manar, 2098 El-Manar II, Tunisia

Nowadays, microorganisms play a remarkable role in promotion of science; it has provided a platform for young and established researchers to interact and learn more about the advanced research in microbiology. Worldwide, microorganisms represent a really thoughtful subject on itself; moreover, it can contribute to the science and sustainable development. Therefore, microorganisms are usually considered as a drug-target. The interactions between drugs, their target and their respective direct effects, are generally the way to be explored. In contrast, the microorganism's responses to antimicrobials drug treatments that contribute to cell death are not as well understood and have proven to be quite complex, involving multiple genetic and biochemical pathways.

Furthermore, microorganisms have been used as tools to explore fundamental life processes by researchers. Due to some advantages (e.g., rapid growth, growth manipulation, easy, and quick culture) microbes are frequently used as research models in different fields. Subsequently, microbes play an important role in the research field of enzyme structure and mode of action, drug invention, cellular regulatory mechanism, energy metabolism, protein synthesis, and so on.

Microorganisms can be used as probiotics sources and are defined as “live microorganisms, which, when administrated in adequate numbers, confer a health benefit to the host”. Lactic acid bacteria (LAB), particularly* Lactobacilli*, are widely used in food production and represent the most common microorganisms employed as probiotics in functional foods [1, 2]. The probiotic concept is gaining more attention worldwide, due to the perceived beneficial effects of these bacteria on human and animal health [2]. These microorganisms can produce exopolysaccharides (EPS) which are long-chain polymers that are used industrially as thickeners, stabilizers, and gelling agents in food products. More recently, they were used as depollution agents and there was a growing interest in their biological functions like antitumor, antioxidant, or probiotic activities [3].* Lactobacillus* strains do not elicit antimicrobial effects because their metabolic production is insufficient or minimal. Considering that* Lactobacillus* have these effects in vitro and that their metabolites may target and play a role in the competitive exclusion of pathogens, this kind of bacteria produce numerous antimicrobial peptides and acids that are good bio preservatives for pickled products [4].

Additionally, the production of antibiotics by microorganisms are the main way used nowadays; in fact, polymyxin E, also called colistin, is an important old antibiotic known for around six decades for treatment of infection caused by Gram-negative pathogens. Later studies showed that colistin can also kill Gram-positive bacteria [5]. Moreover, colistin can be biosynthesized by a multienzyme nonribosomal peptide synthetase system (NRPS) in* Paenibacillus polymyxa* [6]. Other natural types of antibiotics are the Antimicrobial peptides (AMPs) which are abundant and ubiquitous in nature. Microbial killing result of rapid interaction of the AMP with the microbial membrane is leading to membrane disruption, release of cytoplasmic constituents, and a halt to cellular activities. Little work is ongoing concerning peptides from Ghanaian marine invertebrates, but crude peptides of* Galatea paradoxa *and* Patella rustica* have been reported to possess some antimicrobial activity [7].

Moreover, the biological properties of propolis have been established several years ago and include antifungal, antiatherogenic, antioxidant, and antimicrobial activities. High content of polyphenols in Chilean propolis can inhibit the growth of* Streptococcus mutans *and reduce biofilm formation without bactericidal effect [8]. Polyphenols from Chilean have also been shown to affect the expression of genes involved in* S. mutans *virulence and the capacity for forming a biofilm [9].

Plants are another source of natural products which have been largely used in different domains. Aromatic and medicinal plants have been reported to contain a higher content of bioactive phytochemicals such as substantial number of vitamins, phenolic compounds, and essential oils and thus can be used as important sources of natural antioxidants for food application and pharmaceuticals [10]. Recently,* Pelargonium graveolens* is a herb belonging to the family Geraniaceae that has shown good aromatic properties. It is cultivated worldwide, mainly for its essential oil fraction, which is extensively used in various industries. The essential oil of the fresh plant is widely used in perfume industry [11]. Besides, many studies on active molecules in essential oil and organic extracts of* Pelargonium graveolens* have shown good antioxidant activity and antimicrobial effect, specifically against* Bacillus cereus, B. subtilis, *and* Staphylococcus aureus* [12]. However, because of the toxicity of essential oils and organic extracts, their application in food against spoilage pathogens is limited, and more interest in safety matters should be shown [13].

Currently, natural compounds obtained from vegetables with antibacterial properties could be considered as an alternative to conventional antibiotics [14]. In recent years, the antibacterial properties of some compounds obtained from* Allium* plants such as garlic (*Allium sativum*) and onion (*Allium cepa*) have been described. These can inhibit the growth of a range of Gram-positive and Gram-negative bacteria, including both pathogenic and commensal bacteria in humans and animals [15].

Finally, the textile industry is one of the most polluting industries of clean water; recently microorganisms can be used as wastewater treatment alternative. However, bioprocessing can be considered as a preferred option to overcome these disadvantages because it is cost saving and environmentally friendly. Biological treatments can be used to degrade and/or to adsorb azo dyes contaminants [16]. The most efficient microorganisms to break down colored pollutants so far reported are white-rot fungi. These comprise mostly basidiomycetous fungi, which are capable of extensive aerobic lignin degradation and mineralization. This is possible through several extracellular lignin-degrading enzymes, such as lignin peroxidase, manganese-dependent peroxidase, and laccase [17].

This special issue was dedicated to the First International Congress on Biochemistry and Microbiology Applied Technologies “BMAT-2017” which was held in Tunisia, 03-05 November 2017. In this special issue, several full-length papers related to microorganisms as targets and tools in diverse field of studies were published. Furthermore, the current special issues included collected manuscripts from outside the conference provided that they fit within the scope of the special issue.
